# An outbreak of shigellosis in European travellers returning from Cape Verde

**DOI:** 10.1016/j.nmni.2023.101091

**Published:** 2023-01-26

**Authors:** Yash Chandani, Victor Ghosh, Vinay Suresh, Vaibhav Singh, Mubarick Nungbaso Asumah, Bijaya Kumar Padhi

**Affiliations:** King George's Medical University, Lucknow, Uttar Pradesh, India; Andhra Medical College, Visakhapatnam, Andhra Pradesh, India; King George's Medical University, Lucknow, Uttar Pradesh, India; Ministry of Health, Nurses' and Midwives' Training College, P.O. Box 565, Tamale, Northern Region, Ghana; Department of Community Medicine and School of Public Health, Postgraduate Institute of Medical Education and Research, Chandigarh, 160012, India

**Keywords:** Dysentery, Outbreak, Shiga bacillus, Shigella dysentery, Shigella infection, Shigella sonnei, Shigellosis, Sweden, Travellers'risk

## Abbreviations used

EU: European Union
WHO: World Health Organization
HIV: Human Immunodeficiency Virus
CDC: Centers for Disease Control and Prevention
XDR: Extensively drug-resistant
WASH: Water, Sanitation, and Hygiene

Dear Editor:

Shigellosis is a relatively rare condition in the EU. The European Centre for Disease Prevention and Control reported that the notification rate for the disease was 0.7 in the year 2020 [1]. However, the Public Health Agency of Sweden - Folkhälsomyndigheten - has reported that 30 cases of *Shigella* have been brought to notice since mid-November 2022 [2]. The outbreak in Sweden succeeds a paradigm of cases of Shigellosis being reported from several European countries among travellers since August 2022. The increase in cases has been associated with the travel history of the patients to Cape Verde, Africa [2]. 11 bacterial isolates have been identified, out of which nine are of *Shigella sonnei,* and 2 are *Shigella boydii* species. Apart from *Shigella*, other pathogens like *Campylobacter*, *E. coli*, *Giardia,* and *Cryptosporidium* have been noted to cause infections.

### Travellers' risk

**Fig. 1:**
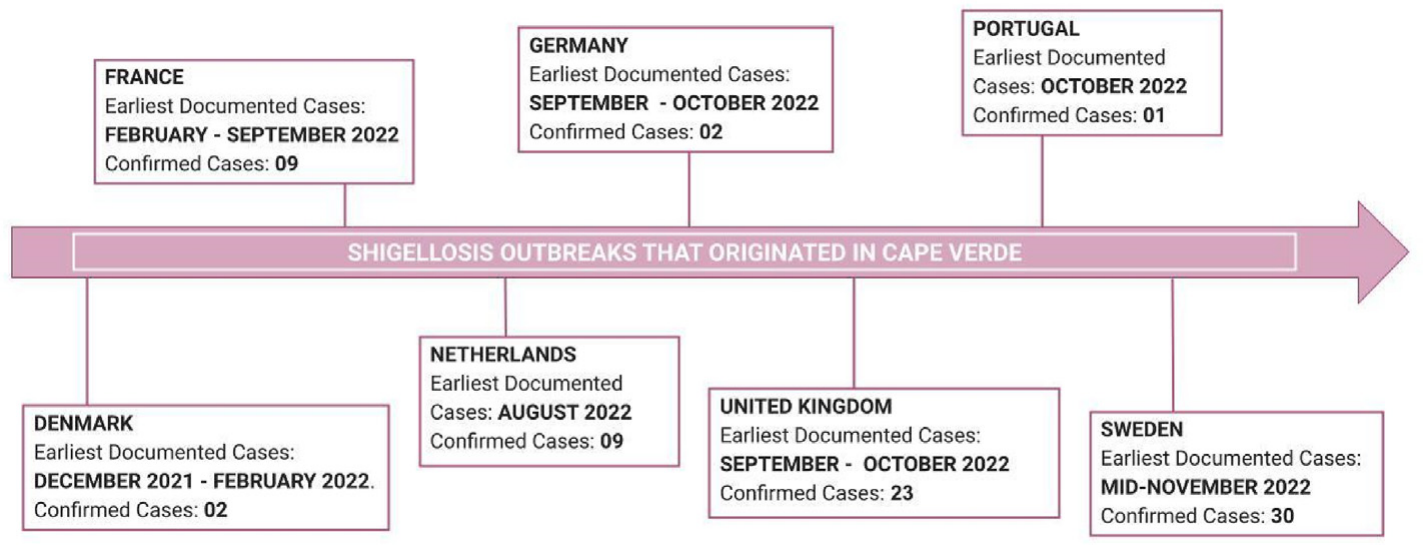
A Timeline highlighting outbreaks of Shigellosis that originated in the islands of Cape Verde and spread to other countries across Europe.

The outbreak of Shigellosis reported in August 2022 in multiple European countries has been linked to a common travel history in Cape Verde, Africa (Fig. 1). The countries affected by *Shigella sonnei* include the Netherlands, Germany, Denmark, France, Portugal, and the UK. Apparently, most of the travellers stayed in the same hotel chain during their visit to Cape Verde. Among the detected cases, resistance has been found against streptomycin, trimethoprim, and hydrogen peroxide [3]. Although the exact cause has not yet been confirmed, the most probable cause is some form of food or water resource contamination at the site.

Unhygienic conditions facilitate the spread of infection and are a concern for travellers visiting such areas. About 2-9% of travellers' diarrhoea is caused due to *Shigella* infection based on fecal cultures [4]. This could be due to the consumption of contaminated food and water or external exposure during recreational activities. Travelling in groups is a common practice, which increases the chances of transmission. Interpersonal contact is the other route of spread, which is most commonly found in men who have sex with men.

As the infection spreads through travellers of multi-racial and multi-national origin, the bacilli naturally adapt to different environments, eventually leading to the emergence of different variants and, ultimately, species. This has also become a major contributor to antimicrobial resistance propagation among different Shigella species. As such, empirical treatment for Shigella cases is progressively being based on the patient's travel history.

As an upside to this largely negative predisposition, the sudden rise of cases in a developed country can be traced back to a developing country through travellers. The utilization of surveillance systems can be used to diagnose patterns of travel history in cases presenting the illness in developed countries. Hence, travellers can be used as sentinels to determine the endemicity of Shigellosis in countries with fewer resources.

### Concerns to be raised

Shigella usually leads to an array of clinical manifestations, such as fever, nausea, and watery diarrhoea. Occasionally, invasive infections such as meningitis, osteomyelitis, arthritis, and splenic abscess occur. *Shigella* sepsis has a bad prognosis and typically affects young, malnourished children as well as HIV-positive individuals [4].

The reporting of cases in non-endemic regions whose origin have been linked to places like Cape Verde is a cause of concern. To add to it, travellers aid in the spread of antimicrobial-resistant bacteria across continents, which can facilitate the emergence of mutant drug-resistant strains. In the past, shigella has developed resistance to new antibiotics within ten years of the drug's introduction [4]. The geographical distribution of XDR S. sonnei is under-reported, which makes it difficult for subsequent investigations and implementation of measures. In order to identify possible introductions of shigella into new regions and prevent local outbreaks in communities, WHO has encouraged national authorities to step up their *Shigella* surveillance and antimicrobial resistance testing [5].

### The way forward

Judicious antimicrobials use, careful history-taking, and efficient contact tracing can contribute to an overall decrease in the disease burden. Despite a century of *Shigella* vaccine research, there is no approved vaccine against Shigellosis. So, other modalities that focus on a preventative approach need investment, such as WASH (Water, Sanitation, and Hygiene) strategies to reduce the burden of enteric infections worldwide. Maintaining hygienic conditions and regular sanitation practices can help prevent transmission. Shigella transmission from index cases to household members has been shown to be 70% reduced by handwashing [4]. Recommended public health control measures should involve the exclusion of Shigellosis patients from food preparation, childcare, and work. Research should focus on the development of a safe and effective vaccine along with advancements in surveillance models to track infected individuals, thereby preventing widespread transmission of the disease.

### Funding

There was no source of funding for this research.

### Ethical approval and informed consent

Ethical approval and informed consent were not required for this study.

### Data availability

Data sharing is not applicable to this article as no new data were created.

### Authors contribution

Equally contributed.

### Conflicts of interest

The authors declare no conflict of interest.
